# Exercise participation and health promotion in active seniors in aging societies: keys to successful aging

**DOI:** 10.3389/fpubh.2025.1635394

**Published:** 2025-11-20

**Authors:** Soo-Jin Choi, Fang Zheng, Seung-Man Lee

**Affiliations:** 1 Department of Education, Zhejiang University, Hangzhou, Zhejiang, China; 2 Education Department, Zhejiang University, Hangzhou, Zhejiang, China; 3 Department of Sports Science, Hankyong National University, Anseong, Republic of Korea

**Keywords:** active seniors, successful aging, health promotion, exercise participation, perceived health, lifestyle behaviors, aging societies

## Abstract

**Introduction:**

As populations age worldwide, promoting health and autonomy in later life has become a shared policy imperative. However, little is known about how concurrent lifestyle behaviors among physically active seniors influence their perceived health outcomes.

**Methods:**

Using data from the 2023 National Survey on Physical Activity, this study analyzed 1,729 physically active older adults in South Korea. Descriptive statistics, independent t-tests, and multiple regression analyses were conducted to examine the associations between exercise participation, co-occurring health behaviors, and self-reported gains related to daily functioning and healthcare costs.

**Results:**

Regular diet management and nutritional supplementation, along with abstinence from alcohol and smoking, were consistently associated with greater perceived assistance in daily activities and reduced healthcare costs. In contrast, regular physical activity and adequate rest showed no significant associations with most positive outcomes, except for a modest link with reduced healthcare costs. Self-reported gains—especially in daily activity assistance and cost reduction—were positively associated with perceived health and fitness status.

**Discussion:**

Among already active seniors, integrated lifestyle strategies emphasizing dietary management and substance abstinence appear to yield more tangible benefits than exercise alone. Policy and practice should therefore complement exercise promotion with nutrition support and cessation programs, leveraging community sports-club infrastructures to sustain these behaviors in aging societies.

## 1 Introduction

### 1.1 Background and context

As populations around the world age rapidly in the 21st century, ensuring the health and well-being of older adults has emerged as a critical public health priority. According to United Nations standards, a society with more than 7% of its population aged 65 years or older is defined as an “aging society,” 14% or more as an “aged society,” and 20% or more as a “super-aged society” or “post-aged society” (1). South Korea is one of the fastest-aging countries globally. According to recent UN and OECD reports, South Korea is on the cusp of becoming a super-aged society, with the share of adults aged 65 + approaching or exceeding 20% (1, 2). These demographic changes pose challenges at individual, family, community, and national levels, necessitating systematic approaches to maintain health and improve quality of life among older adults. Globally, the World Health Organization (3) and OECD (2) have reported similar demographic shifts in Europe, North America, and Japan, highlighting the economic and healthcare challenges of supporting older populations. These comparative insights emphasize that the Korean case reflects a broader global imperative to promote successful aging. Prior consensus statements also emphasize cardiovascular evaluation in aging populations (4).

### 1.2 Emergence of active seniors and relevance of exercise

In response to these challenges, a new generation called “active seniors” is gaining attention. Coined by Bernice Neugarten, a professor at the University of Chicago in the United States, the term reflects the idea that today’s seniors are not the same as yesterday’s seniors. Active seniors are those who pursue healthy and active lives by enthusiastically participating in social, cultural, and physical activities, even after retirement. In Korea, the emergence of active seniors is attributed to the economic growth of the baby boom generation, higher education levels, and advancements in medical technology, which enable them to seek active and proactive lives for self-actualization. Comparative studies also note that in Western Europe, Japan, and North America, active seniors increasingly participate in lifelong learning, volunteering, and organized sports, suggesting that the phenomenon is culturally widespread but expressed differently across societies.

Exercise participation is widely recognized as a key component of healthy aging. Research highlights the role of exercise in promoting physical health, mental well-being, and social connections. Group-based exercise programs also help reduce isolation and support independence among older adults. Consequently, exercise participation is considered one of the most effective strategies for achieving successful aging (5). These mechanisms align with established theories of exercise behavior (6), and sedentary lifestyles have long been linked to chronic disease risk (7).

### 1.3 Research gap

Despite a growing body of research on exercise participation among older adults, studies specifically focusing on active seniors remain limited. Most prior studies treat older adults as a homogeneous population without distinguishing those who are already engaged in health-promoting behaviors. Furthermore, few studies have examined how multiple health behaviors—including diet, rest, and substance abstinence—interact with exercise participation to influence perceived health and fitness outcomes. There is also limited discussion on how such findings can be translated into practical policy measures and targeted interventions for active seniors in rapidly aging contexts such as South Korea.

### 1.4 Contribution of this study

This study addresses these gaps by analyzing the relationships between exercise participation, comprehensive health maintenance behaviors, and perceived health outcomes among 1,729 active seniors using data from the 2023 National Survey on Physical Activity conducted by the Ministry of Culture, Sports, and Tourism. By examining differences according to gender and sports club membership, the study extends current understandings of how demographic and behavioral factors interact to shape health perceptions. Moreover, by highlighting the influence of integrated health behaviors—including diet, rest, and substance abstinence—this research provides evidence-based insights for designing community-level health promotion strategies.

### 1.5 Practical implications

These findings have the potential to inform public health initiatives and policy decisions. In particular, they can guide the development of tailored health strategies, community programs, and educational campaigns aimed at enhancing health, reducing healthcare costs, and supporting successful aging in super-aged societies.

### 1.6 Conceptual framework

**Figure 1:**
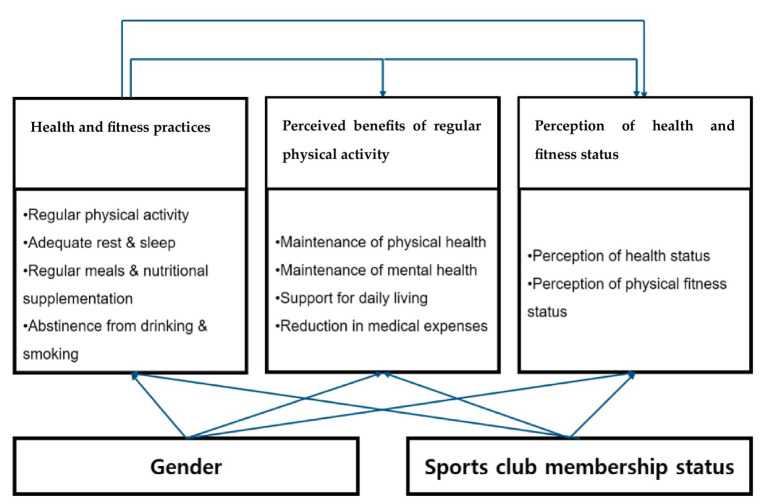
Conceptual framework of the study. Health and fitness maintenance behaviors shape the perceived benefits of regular physical activity, which subsequently influence perceived health and fitness status.

The research model, grounded in Fishbein and Ajzen’s Theory of Reasoned Action (8), illustrates how health and fitness maintenance behaviors influence practical benefits of regular physical activity, which in turn affect perceived health and fitness status. Figure 1 presents this conceptual framework and is provided here to orient the reader before the Methods section.

## 2 Methods

### 2.1 Data source and survey design

This study utilized data from the 2023 National Survey on Physical Activity conducted by the Ministry of Culture, Sports, and Tourism (MCST), Korea (9). The MCST conducts this annual survey to understand nationwide participation in physical activities, develop environments conducive to participation, and provide evidence for policy decisions aimed at increasing engagement and satisfaction. The survey is a nationally approved statistic (No. 113003, October 29, 1991) under Article 33 of the Statistics Act. In 2023, a stratified random sample of 9,000 Korean citizens aged 10 years or older was surveyed between August and November 2023. Detailed procedures for survey design, sampling, and data processing are outlined in Table 1 of this study. All procedures conformed to ethical standards and were approved as exempt from review by the Institutional Review Board of the Korea National University of Education (KNUE-202409-SB-0562-01).

**Table 1:** Research contents.

Steps	Calendar	Contents
Preparing investigation	July–August 2023	⦁Initiate reporting ⦁Survey design and questionnaire consultation ⦁Discuss analytics
Collecting data	September–October 2023	⦁Leverage tract information to build representative populations ⦁Draw a sample population ⦁Select, train, and manage surveyors ⦁Create research guides and editorial guides ⦁Create the tools you need for your survey, including purchasing survey tools and response examples. ⦁Conduct a survey
Data processing and validation	October–November 2023	⦁Three rounds of edits during the research period ⦁Questionnaire aggregation and data entry ⦁Data aggregation error validation ⦁Data cleaning
Analyzing and publishing results	December 2023– January 2024	⦁Create and submit final results reports ⦁Register reports and resources homepage ⦁ Issue a press release

### 2.2 Participants

For the purpose of this study, a subset of 1,729 individuals in their 50s and 60s who regularly engaged in physical activities and met the criteria for being considered active seniors were analyzed. Participant demographic information, including gender, age, educational level, marital status, household composition, income level, residential area, and sports club membership, was collected and is presented in Table 2. In the Korean aging policy context, planning research on welfare housing for active seniors highlights autonomy and participation in later life (10).

**Table 2:** General characteristics of participants.

Variables	Separation	Main investigation	
		Number of cases	Percentage (%)
Gender	Male	844	48.8
	Female	885	51.2
Age	50	1,048	60.5
	60s	681	39.4
Education	Graduated from elementary school	12	0.7
	Graduated from middle school	77	4.4
	Graduated from high school	884	51.1
	Graduated from community college	328	19.0
	Graduated from a four-year college	410	23.7
	Master’s degree	13	0.8
	PhD	5	0.3
Marital status	Married	1,637	94.6
	Single	12	0.7
	Bereavement	29	1.7
	Divorce	51	2.9
Number of people in the household	1 person	65	3.8
	2 people	525	30.3
	3 people	532	30.7
	4 people	563	32.5
	5 people	44	2.5
Child status	None	73	4.2
	1	436	25.2
	2	1,028	59.4
	3	180	10.4
	4 or more	12	0.7
Monthly household income	Less than 1 million	6	0.3
	1–1.99 million	58	3.4
	2–2.99 million	179	10.3
	3–3.99 million	312	18.0
	4–4.99 million	399	23.1
	5–5.99 million	335	19.4
	6–6.99 million	250	14.4
	7–7.99 million	111	6.4
	8–8.99 million	43	2.5
	Over 9 million	36	2.1
Where you live (City size)	Large cities	760	43.9
	Small and medium-sized cities	583	33.7
	Town or less	386	22.3
Athletic club membership	Signed up	301	17.4
	Not signed up	1,428	82.6
All		1,729	100

### 2.3 Research model and variable selection

The research model was developed based on the Theory of Reasoned Action proposed by Ajzen and Fishbein (8), which emphasizes that attitudes and beliefs drive behavioral intentions and actual behaviors. Informed by prior studies on health behavior and aging (5, 6), four key constructs were included: health and fitness maintenance behaviors (regular physical activity, adequate rest and sleep, regular diet and nutritional supplementation, and abstinence from alcohol and smoking); perceived advantages of regular physical activity (staying physically fit, staying mentally healthy, assisting daily activities, and reducing healthcare costs); perceived health status; and perceived fitness status (Tables 3–6).

**Table 3:** Descriptive statistics (average scores out of 5.0).

Variables		Average	Standard deviation	Skewness	Kurtosis
Performing health and fitness maintenance	Regular physical activity	3.55	0.769	−0.141	0.129
	Get enough rest and sleep	3.65	0.694	−0.543	0.370
	Eat regularly and supplement with nutrition	3.69	0.731	−0.261	0.033
	Abstinence from alcohol and smoking	3.59	1.060	−0.382	−0.698
	TOTAL	3.62	0.533	−0.498	0.421
Benefits of regular physical activity	Stay physically fit	4.03	0.547	−0.067	0.663
	Staying mentally healthy	4.15	0.610	−0.154	−0.163
	Help with Everyday life	3.79	0.691	−0.050	−0.244
	reduce healthcare costs	3.60	0.679	−0.130	−0.093
	TOTAL	3.89	0.454	−0.173	0.169
Recognize health and fitness status	Perceived health status	3.70	0.610	−0.636	0.640
	Recognizing health status	3.54	0.692	−0.296	−0.046

Tested using frequency analysis.

**Table 4:** Correlations between variables.

Variables	A	B	C	D
A	1	–	–	–
B	0.291***	1	–	–
C	0.313***	0.172***	1	–
D	0.332***	0.184***	0.612***	1
*M* ± SD	3.618 ± 0.533	3.891 ± 0.454	3.700 ± 0.610	3.540 ± 0.692

A: Health and fitness maintenance behavior, B: Effects of regular physical activity, C: Perceived health status, D: Perceived fitness status; Tested using correlation analysis; ***Correlation coefficients are significant at the 0.001 level (two-tailed).

**Table 5:** Gender differences in health and fitness maintenance behaviors, perceived benefits of regular physical activity, and perceived health/fitness status (*N* = 1,729).

Variable	*M* ± SD		*T*	*p*
	Male	Female		
Regular physical activity	3.52 ± 0.783	3.58 ± 0.755	−1.641	0.101
Adequate rest and sleep	3.65 ± 0.690	3.64 ± 0.697	0.26	0.795
Regular diet and nutrition	3.65 ± 0.748	3.72 ± 0.715	−1.837	0.066
Abstinence from alcohol and smoking	3.16 ± 1.010	4.00 ± 0.934	−18.075	< 0.001^***^
Perceived health status	3.76 ± 0.599	3.64 ± 0.616	4.261	< 0.001^***^
Perceived fitness status	3.63 ± 0.668	3.45 ± 0.703	5.51	< 0.001^***^
Perceived benefit: staying physically fit	4.04 ± 0.557	4.02 ± 0.538	0.712	0.476
Perceived benefit: staying mentally healthy	4.14 ± 0.611	4.16 ± 0.609	−0.661	0.509
Perceived benefit: Assisting daily activities	3.79 ± 0.676	3.78 ± 0.705	0.357	0.721
Perceived benefit: reducing healthcare costs	3.60 ± 0.700	3.60 ± 0.658	0.051	0.959

Tested using independent samples *t*-test; ***p < 0.001.

**Table 6:** Differences in health behaviors, perceived benefits, and perceived health/fitness status by sports club membership (*N* = 1,729).

Variable	*M* ± SD		*T*	*p*
	Members	Non-members		
Regular physical activity	3.71 ± 0.688	3.66 ± 0.623	4.359	<0.001^***^
Adequate rest and sleep	3.75 ± 0.663	3.62 ± 0.698	3.085	0.002^**^
Regular diet and nutrition (with Supplementation)	3.92 ± 0.683	3.64 ± 0.732	6.464	<0.001^***^
Abstinence from alcohol and smoking	3.40 ± 1.108	3.63 ± 1.046	−3.328	<0.001^***^
Perceived health status	4.19 ± 0.604	4.00 ± 0.529	5.049	<0.001^***^
Perceived fitness status	4.31 ± 0.602	4.12 ± 0.607	5.074	<0.001^***^
Perceived benefit: staying physically fit	3.84 ± 0.654	3.78 ± 0.698	1.556	0.120
Perceived benefit: staying mentally healthy	3.68 ± 0.733	3.58 ± 0.666	2.481	0.020^*^

Tested using independent samples *t*-test; *p < 0.05, **p < 0.01, ***p < 0.001.

These variables were selected because prior literature consistently identifies them as significant determinants of successful aging and health promotion among older adults (6). Their inclusion allows for comprehensive examination of how integrated lifestyle behaviors interact with exercise participation to influence self-reported health outcomes.

### 2.4 Measurement tools

Nineteen items from the 2023 National Survey on Physical Activity were utilized as research instruments. Demographic variables were measured on a nominal scale. Health and fitness maintenance behaviors, practical benefits of regular physical activity, and perceived health and fitness status were assessed using a 5-point Likert scale, with higher scores indicating more favorable behaviors or perceptions. Reliability and validity of these items were established in the original national survey, and therefore additional testing was not required. However, it should be acknowledged that no revalidation was conducted within the current study context, which constitutes a methodological limitation. Future studies should consider confirmatory factor analyses or other validation procedures to enhance measurement rigor. For context, objective functional assessments commonly used in aging research—such as the Short Physical Performance Battery (SPPB)—offer benchmarks for lower-extremity function, disability, and institutionalization risk, although they were not implemented in the present study (11).

### 2.5 Data analysis

Data were analyzed using SPSS 24.0 (IBM Corp., Armonk, NY, United States). Frequency analyses were conducted to examine participant characteristics. Descriptive statistics and correlation analyses were used to assess the normality of the data and relationships between variables. Independent samples *t*-tests were performed to examine differences in variables according to gender and sports club membership. Multiple regression analyses were employed to investigate the influence of health and fitness maintenance behaviors on health-related advantages of regular physical activity, and subsequently, the effect of practical benefits on perceived health and fitness status. The Durbin–Watson statistic was applied to confirm the absence of autocorrelation in residuals for each regression model. While only parametric tests were applied in the present study, future research should also consider non-parametric approaches to account for potential deviations from normality and to ensure robustness of results.

## 3 Results

### 3.1 Descriptive statistics and correlations

Descriptive statistics for health and fitness maintenance behaviors, positive outcomes of regular physical activity, and perceived health and fitness status indicated mean scores ranging from 3.54 to 4.15, with standard deviations from 0.454 to 1.060. Skewness and kurtosis values met the criteria for normality (skewness < ±3.0, kurtosis < ±10.0), supporting the use of parametric tests. Correlation analyses demonstrated positive associations among all variables, with coefficients ranging from 0.172 to 0.612, confirming no multicollinearity issues. These descriptive results suggest that active seniors in this sample generally reported high engagement in health-promoting behaviors and favorable health perceptions, which provides important context for interpreting subsequent group comparisons and regression models.

### 3.2 Gender differences in variables

Independent samples *t*-tests revealed notable gender differences in certain health behaviors and perceptions. Women scored significantly higher than men in abstaining from alcohol and smoking (*p* < 0.001). However, no significant gender differences were found in regular physical activity, adequate rest, or regular diet and nutrition. In terms of perceived health and fitness status, men reported significantly higher health status and fitness status perceptions than women (*p* < 0.001). No significant gender differences were observed in the reported gains of regular physical activity. These findings are consistent with sociocultural patterns observed in Korea and elsewhere: women in mid-to-later life often adopt more risk-avoidant behaviors such as avoiding alcohol and smoking, while men tend to rate their own health status more positively despite similar or poorer objective conditions. This highlights the need for gender-sensitive approaches to health promotion among older adults. Social isolation has further been identified as a major risk factor for depressive symptoms among older adults (12).

### 3.3 Differences according to sports club membership

Participants who were members of sports clubs reported significantly higher scores for regular physical activity, adequate rest and sleep, and regular meals and nutrition than nonmembers. Interestingly, nonmembers scored higher in abstaining from alcohol and smoking. Members also reported significantly greater advantages of regular physical activity (staying physically fit, staying mentally healthy, and reducing healthcare costs) and higher perceived health and fitness status compared with nonmembers. These results suggest that participation in organized sports clubs not only promotes consistent health behaviors but also strengthens perceived benefits and self-rated health among seniors. From a policy perspective, expanding community-based sports club access could be a key lever for sustaining health-promoting behaviors in aging societies. Beyond Korea, out-of-home physical activity is associated with lower depressive symptoms among older adults (12), and qualitative studies highlight football clubs as valuable platforms for health promotion (13).

### 3.4 Impact of health and fitness practices on practical benefits

Multiple regression analyses revealed that regular diet and nutritional supplementation had significant positive effects on staying physically fit, staying mentally healthy, assisting daily activities, and reducing healthcare costs (all *p* < 0.001). Abstinence from alcohol and smoking also had significant positive effects on assisting daily activities and reducing healthcare costs (*p* < 0.001). Regular physical activity and adequate rest and sleep showed no significant effects on most practical gains variables. One possible explanation is a “ceiling effect,” given that the sample already consisted of individuals with high levels of activity and generally good health practices. Additionally, measurement limitations—such as reliance on self-reported behaviors—may have contributed to the non-significant findings. This suggests that their influence may be more indirect, shaping global health perceptions rather than day-to-day functional gains. Nutritional supplementation interventions have also demonstrated reductions in re-hospitalization and medical costs (14). Randomized trials show that structured nutritional support reduces readmissions and improves outcomes in older patients (14, 15). Regular physical activity and adequate rest and sleep showed no significant effects on most practical gains variables. However, in regression models predicting overall perceived health and fitness status (Table 7), both variables emerged as significant predictors.

**Table 7:** Regression analyses of health and fitness maintenance behaviors predicting perceived benefits of regular physical activity (*N* = 1,729).

Dependent variable	Independent variables	*B*	S. E.	β	*t*	*p*	Statistics
Stay physically fit	Constant	3.384	0.092	–	36.618	<0.001^***^	*R* = 0.196 *R*^2^ = 0.039 Adjusted *R*^2^ = 0.036 *F* = 17.260, *p* < 0.000^***^
	Regular physical activity	0.024	0.017	0.033	1.380	0.168	
	Get enough restful sleep	0.027	0.023	0.034	1.156	0.248	
	Eat nutritional supplements regularly	0.124	0.022	0.166	5.573	<0.001^***^	
	Abstinence from alcohol and smoking	0.002	0.013	0.003	0.124	0.901	
Stay mentally healthy	Constant	3.457	0.102	–	33.821	0.000^***^	*R* = 0.231 *R*^2^ = 0.054 Adjusted *R*^2^ = 0.051 *F* = 24.390, *p* < 0.000^***^
	Regular physical activity	−0.011	0.019	−0.013	−0.562	0.574	
	Get enough restful sleep	0.042	0.026	0.047	1.627	0.104	
	Eat nutritional supplements regularly	0.174	0.025	0.209	7.056	<0.001^***^	
	Abstinence from alcohol and smoking	−0.017	0.014	−0.029	−1.194	0.233	
Help with daily activities	Constant	2.647	0.114	–	23.166	<0.001^***^	*R* = 0.278 *R*^2^ = 0.077 Adjusted *R*^2^ = 0.075 *F* = 36.087, *p* < 0.000^***^
	Regular physical activity	0.014	0.021	0.016	0.664	0.507	
	Get enough restful sleep	0.042	0.029	0.042	1.469	0.142	
	Eat nutritional supplements regularly	0.185	0.028	0.196	6.698	<0.001^***^	
	Abstinence from alcohol and smoking	0.071	0.016	0.109	4.513	<0.001^***^	
Reduce healthcare costs	Constant	2.437	0.113	–	21.566	<0.001^***^	*R* = 0.256 *R*^2^ = 0.066 Adjusted *R*^2^ = 0.063 *F* = 30.279, *p* < 0.000^***^
	Regular physical activity	0.070	0.021	0.079	3.340	<0.001^***^	
	Get enough restful sleep	0.044	0.028	0.045	1.542	0.123	
	Eat nutritional supplements regularly	0.145	0.027	0.156	5.317	<0.001^***^	
	Abstinence from alcohol and smoking	0.061	0.016	0.095	3.886	<0.001^***^	
Perceived health status	Constant	2.191	0.097	–	22.510	<0.001^***^	*R* = 0.378 *R*^2^ = 0.143 Adjusted *R*^2^ = 0.141 *F* = 71.798 *P* < 0.000^***^
	Regular physical activity	0.116	0.018	0.146	6.430	<0.001^***^	
	Get enough restful sleep	0.117	0.024	0.133	4.815	<0.001^***^	
	Eat nutritional supplements regularly	0.201	0.023	0.241	8.562	<0.001^***^	
	Abstinence from alcohol and smoking	−0.021	0.013	−0.037	−1.572	0.116	
Perceived fitness status	Constant	1.654	0.108	–	15.338	<0.001^***^	*R* = 0.426 *R*^2^ = 0.181 Adjusted *R*^2^ = 0.179 *F* = 95.497 *p* < 0.000^***^
	Regular physical activity	0.149	0.020	0.165	7.461	<0.001^***^	
	Get enough restful sleep	0.225	0.027	0.226	8.331	<0.001^***^	
	Eat nutritional supplements regularly	0.193	0.026	0.204	7.416	<0.001^***^	
	Abstinence from alcohol and smoking	−0.050	0.015	−0.076	−3.324	<0.001^**^	

Tested using multiple regression analysis; *p < 0.05, **p < 0.01, ***p < 0.001.

### 3.5 Impact of reported gains on health and fitness status

Regression results showed that self-reported outcomes such as helping with daily activities and reducing healthcare costs had significant positive effects on perceived health status (*p* < 0.001) and fitness status (*p* < 0.01), whereas staying physically fit and staying mentally healthy did not show significant direct effects. Detailed regression results are provided in Table 8, which is included in the tables section at the end of this manuscript.

**Table 8:** Regression analyses predicting perceived health and fitness status from perceived benefits of regular physical activity (*N* = 1,729).

Dependent variable	Independent variables	*B*	S. E.	β	*T*	*P*	Statistics
Perceived health status	Constant	2.191	0.097	–	22.510	<0.001^***^	*R* = 0.378 *R*^2^ = 0.143 Adjusted *R*^2^ = 0.141 *F* = 71.798 *P* < 0.000^***^
	Regular physical activity	0.116	0.018	0.146	6.430	<0.001^***^	
	Get enough restful sleep	0.117	0.024	0.133	4.815	<0.001^***^	
	Eat nutritional supplements regularly	0.201	0.023	0.241	8.562	<0.001^***^	
	Abstinence from alcohol and smoking	−0.021	0.013	−0.037	−1.572	0.116	
Perceived fitness status	Constant	1.654	0.108	–	15.338	<0.001^***^	*R* = 0.426 *R*^2^ = 0.181 Adjusted *R*^2^ = 0.179 *F* = 95.497 *P* < 0.000^***^
	Regular physical activity	0.149	0.020	0.165	7.461	<0.001^***^	
	Get enough restful sleep	0.225	0.027	0.226	8.331	<0.001^***^	
	Eat nutritional supplements regularly	0.193	0.026	0.204	7.416	<0.001^***^	
	Abstinence from alcohol and smoking	−0.050	0.015	−0.076	−3.324	<0.001^**^	
Perceived health status	Constant	2.890	0.130	–	22.269		*R* = 0.185 *R*^2^ = 0.034 Adjusted *R*^2^ = 0.032 *F* = 15.329 *P* < 0.000^***^
	Staying physically healthy	0.023	0.030	0.021	0.765		
	Staying mentally healthy	0.010	0.027	0.010	0.385		
	Help with everyday life	0.085	0.024	0.096	3.506		
	Reduce healthcare costs	0.096	0.024	0.107	3.979		
Perceived fitness status	Constant	2.605	0.146	–	17.797		*R* = 0.209 *R*^2^ = 0.044 Adjusted *R*^2^ = 0.042 *F* = 19.726 *P* < 0.000^***^
	Staying physically healthy	−0.030	0.034	−0.024	−0.879		
	Staying mentally healthy	0.038	0.030	0.034	1.262		

Tested using multiple regression analysis; *p < 0.05, **p < 0.01, ***p < 0.001.

## 4 Discussion

This study aimed to examine how health and fitness maintenance behaviors are associated with advantages of regular physical activity and, in turn, how these benefits influence perceived health and fitness status among active seniors. By consolidating multiple dimensions of health-related behaviors and perceptions, this research provides a more integrative understanding of factors contributing to healthy aging.

### 4.1 Key findings and interpretation

This study investigated how integrated health behaviors affect perceived benefits of regular physical activity and, ultimately, seniors’ health and fitness status. The results revealed meaningful gender differences in specific behaviors and perceptions. Women reported greater abstinence from alcohol and smoking, while men reported higher perceived health and fitness status. These gender-based patterns are consistent with prior international evidence indicating that women tend to adopt more risk-avoidant lifestyles in later life, whereas men often evaluate their own health more positively despite equal or poorer clinical outcomes (16).

Differences according to sports club membership were also evident. Club members exhibited higher levels of regular physical activity, adequate rest, and nutritional management, as well as stronger reported gains and more positive health and fitness perceptions than non-members. Comparable findings have been reported in studies from Europe and Japan, where organized sports clubs function as important community infrastructures for sustaining healthy lifestyles in older age (13, 17). Additionally, patterns of alcohol and smoking in later life remain relevant for engagement and outcomes (18). From a public health perspective, these findings support expanding access to community-based exercise programs and sports clubs.

Regression analyses demonstrated that among health and fitness maintenance behaviors, regular diet and nutritional supplementation exerted significant positive effects across most advantage domains, while abstaining from alcohol and smoking selectively enhanced specific benefits. Regular physical activity and adequate rest, although fundamental, showed no significant predictive effects for most practical benefits. However, consistent with the regression results (Tables 7, 8), they did show significant associations with overall perceived health and fitness. This non-significance was limited to practical benefit outcomes, whereas for broader perceptions of health and fitness, significant effects were observed (Table 7). This may be explained by a ceiling effect in an already active population, as well as limitations of self-reported survey measures that may underestimate subtle differences in behaviors. Notably, physical activity and adequate rest showed a mixed pattern of effects. They did not significantly predict practical daily-life outcomes such as healthcare cost reduction or assistance with daily activities. However, regression models predicting perceived health and fitness status revealed significant associations (Tables 7, 8). This indicates that while these behaviors may not yield immediate economic or functional advantages within already active seniors, they remain salient for shaping broader perceptions of health and vitality.

Finally, reported gains related to assisting daily activities and reducing healthcare costs significantly predicted perceived health and fitness status, whereas perceived benefits such as staying physically fit or mentally healthy did not directly predict these outcomes. This nuanced finding suggests that seniors may value practical benefits—like cost reduction and daily independence—more strongly than general notions of fitness or mental well-being, a result echoed in recent OECD and WHO reports on aging policy priorities (2, 3). Similar associations between integrated lifestyle behaviors and older adults’ health perceptions have also been reported in international contexts, including longitudinal studies in Europe and North America, underscoring the global relevance of these findings (13, 17).

### 4.2 Theoretical contributions

This study advances existing literature by integrating multiple health maintenance behaviors with self-reported improvements and outcomes in a single model. Unlike prior research that often examined one behavior or benefit in isolation, this analysis demonstrates how diverse behaviors jointly shape perceptions of health and fitness. By incorporating evidence from international studies, the findings strengthen the theoretical generalizability of the active senior concept across cultural contexts.

### 4.3 Practical implications

The findings have direct implications for aging and public health. Interventions promoting healthy aging should emphasize dietary management and substance abstinence alongside physical activity. Updated nutritional guidelines underscore the importance of tailored interventions for older adults (19), and systematic reviews confirm the effectiveness of structured smoking cessation programs (20). Specifically, policy recommendations include: (a) nutritional support programs tailored to older adults, (b) public campaigns for smoking and alcohol cessation, (c) subsidies or incentives for joining community sports clubs, and (d) integration of exercise promotion with broader health education. Policymakers and practitioners should consider strategies that facilitate access to organized sports clubs, which may serve as effective platforms for sustaining these behaviors. Highlighting tangible benefits—such as reduced healthcare costs and improved daily functioning—could further enhance motivation among older adults. Recent WHO guidance recommends multicomponent physical activity—including aerobic, strength, and balance training—tailored to functional capacity in older adults (21, 22). At the systems level, the WHO Global Status Report on Physical Activity calls for scaling community-delivered programs and integrated policies, aligning with our recommendation to expand access to sports clubs (23).

### 4.4 Limitations and future directions

This study is cross-sectional, limiting causal inference. Self-reported measures may introduce response bias, and cultural or regional factors could influence generalizability. Importantly, the exclusive reliance on a Korean sample restricts external validity. Comparative cross-national studies are needed to test whether these findings hold in other cultural settings, such as Western Europe or North America. Future research should also incorporate longitudinal designs, objective health indicators, and qualitative approaches to enrich understanding of subjective perceptions underlying these behaviors and benefits. Evidence from community-based complex interventions indicates meaningful benefits for healthy aging, although heterogeneity across trials suggests careful tailoring and implementation research remain essential (24).

## 5 Conclusion

This study demonstrates that among active seniors, specific health and fitness maintenance behaviors—particularly nutritional management and abstinence from alcohol and smoking—significantly shape reported gains of regular physical activity, which in turn influence perceived health and fitness status. By consolidating multiple behavioral dimensions and highlighting both theoretical contributions and practical applications, this research offers valuable insights for promoting healthy aging.

For policymakers and practitioners, the findings underscore the need to move beyond physical activity alone. Practical strategies should include accessible nutritional support programs, targeted smoking and alcohol cessation initiatives, and expanded opportunities for community-based sports club participation. Such interventions can directly improve daily functioning and reduce healthcare costs, thereby empowering older adults to maintain independence.

For researchers, the study highlights the importance of examining integrated health behaviors and health advantages across different cultural contexts. Future investigations should prioritize longitudinal and cross-national designs to enhance external validity and inform global policy discussions.

In sum, this study not only advances theoretical understanding of successful aging but also calls for immediate, concrete action from policymakers, community leaders, and researchers to design and implement holistic strategies that support active seniors in rapidly aging societies. Consistent with our findings, exercise-based fall-prevention programs deliver population-level benefits among community-dwelling older adults and merit routine integration into community offerings (25). Although dietary management and abstinence behaviors showed the strongest and most consistent effects, physical activity and adequate rest also contributed positively to perceived overall health and fitness, even if their practical benefits were less evident.

## Data Availability

Publicly available datasets were analyzed in this study. This data can be found: the dataset (2023 National Survey on Physical Activity) is managed by the Korea Institute of Sport Science and is available upon request. Currently, there is no public repository or direct link for unrestricted access.
